# Assessing implementation fidelity of an on-site pharmacist intervention within Australian residential aged care facilities: A mixed methods study

**DOI:** 10.1186/s12913-023-10172-9

**Published:** 2023-10-27

**Authors:** Miranda Batten, Jane Koerner, Sam Kosari, Mark Naunton, Joanne Lewis, Karen Strickland

**Affiliations:** 1 Health Research Institute, University of Canberra, Bruce, ACT 2617 Australia; 2 Discipline of Pharmacy, Faculty of Health, University of Canberra, Bruce, ACT 2617 Australia; 3 School of Nursing and Health, Avondale University, Wahroonga, NSW 2076 Australia; 4 School of Nursing, Midwifery and Public Health, University of Canberra, Bruce, ACT 2617 Australia; 5 School of Nursing and Midwifery, Edith Cowan University, Joondalup, WA 6207 Australia

**Keywords:** Implementation fidelity, Pharmacist, Aged care, Intervention delivery, On-site pharmacist

## Abstract

**Background:**

An on-site pharmacist (OSP) intervention was implemented which sought to improve medication management within residential aged care facilities (RACFs) in the Australian Capital Territory, Australia. The objectives of this mixed methods study were to evaluate the implementation fidelity of the OSP intervention and to determine the moderating factors which influenced delivery of this intervention.

**Methods:**

This convergent parallel mixed methods study was underpinned by Hasson’s conceptual framework for implementation fidelity. Implementation fidelity for seven intervention RACFs was quantitatively assessed using three quantitative data sets: (1) range of OSP intervention activities delivered; (2) random sample of 10% of medication reviews assessed for quality; (3) proportion of residents who received at least one medication review. Semi-structured interviews (n = 14) with managers and OSPs across the intervention RACFs were conducted to identify moderating factors which may have influenced OSP intervention delivery.

**Results:**

The OSP intervention was generally delivered as intended with overall medium levels of implementation fidelity. This delivery was supported by a range of facilitation strategies with most participants perceiving that the intervention was delivered to a high standard. RACF managers and OSPs were mostly well engaged and responsive. A number of potential barriers (including the part-time OSP role, COVID-19 pandemic, RACFs spread out over a large area with significant distance between resident dwellings) and facilitators (including the pharmacist support meetings, OSPs who took time to establish relationships, RACF managers who actively supported OSPs and worked with them) for OSP intervention delivery were identified which have potential implications for the roll out of OSPs within Australian RACFs.

**Conclusion:**

In this study, the implementation fidelity of OSP intervention delivery was assessed with overall medium levels of fidelity found across the intervention RACFs. This suggested that the OSP intervention can generally be delivered as intended in real world RACFs. OSP intervention delivery was influenced by a range of moderating factors, some of which posed barriers and others which facilitated the OSP intervention being delivered as intended.

**Supplementary Information:**

The online version contains supplementary material available at 10.1186/s12913-023-10172-9.

## Introduction

### Background

Residents living in Residential Aged Care Facilities (RACFs) are at high risk of medication-related problems [1] which can lead to medication-related harm. Medication-related harm is the overarching term used to describe harm amongst patients caused by medication errors and unsafe medication practices ranging from prescribing of potentially inappropriate medication through to dispensing and administration errors [2]. Internationally, inappropriate medication use impacts around 50% of residents living in RACFs [3] and it has been suggested that 95% of residents living in Australian RACFs have at least one-medication related problem [4]. Residents living in RACFs are also more likely to be prescribed potentially inappropriate medications compared to older people living at home [5–7] which may increase their risk of experiencing medication-related harm.

The importance of improving medication management^1^ processes to potentially help reduce medication-related harm is illustrated by the inclusion of medication management – polypharmacy and medication management – antipsychotic as quality indicators by the Aged Care Quality and Safety Commission [8]. This means that medication management is recognised as an important quality of care aspect which has the potential to impact upon the health and wellbeing of residents living in Australian RACFs [8]. There have been ongoing efforts to improve medication management within Australian RACFs. A 2017 pilot study conducted in Canberra, Australian Capital Territory (ACT) identified some promising findings associated with having an on-site pharmacist (OSP) working in a RACF [9]. Following on from this pilot study, in 2020, an OSP intervention was implemented as part of a cluster randomised controlled trial (RCT) in RACFs in Canberra, ACT [10]. The *Pharmacists in Residential Aged Care Facilities* (PiRACF) study evaluated the effectiveness and implementation of a 12-month OSP intervention which sought to improve medication management.

Implementation fidelity is commonly described as the extent to which an intervention was delivered as intended [11]. Implementation fidelity may be considered a core process evaluation component [12] or an aspect of implementation [13]. Historically, implementation fidelity has been seldom assessed when evaluating pharmacist interventions in health care settings [13, 14] but has begun to change recently [15–19]. Assessment of implementation fidelity can help inform whether the intervention’s outcome was due to design issues (i.e. theory failure) or intervention delivery issues (i.e. implementation failure), thus supporting real-time intervention delivery modifications and adoption of the intervention [20].

This mixed methods study was conducted within the context of the PiRACF study to understand the extent to which the OSP intervention was delivered as intended and determine the factors which influenced this intervention delivery across the seven intervention RACFs. This increased understanding has the potential to optimise the roll out of OSPs in Australian RACFs by determining whether the OSP intervention can be delivered as intended in real world RACFs. The identification of potential barriers and facilitators to successful OSP intervention delivery is also timely given that the Australian Government will be funding OSPs in RACFs from 2023 [21].

### Aim

The aim of this study was to evaluate the implementation fidelity of the OSP intervention and understand the moderating factors which may have impacted delivery of the OSP intervention.

## Methods

### Study design

This study’s focus on intervention delivery is consistent with Gearing et al.’s assertion that intervention delivery is a core aspect of implementation fidelity [22]. The use of mixed methods study design for this study is consistent with previous health care implementation fidelity studies [17–19, 23–26]. A convergent parallel design was employed in this study wherein the quantitative and qualitative data were collected and analysed separately and then merged and integrated [27, 28]. This approach helps to offset any potential weaknesses associated with the individual data sets. The use of an existing implementation fidelity framework is also consistent with previous health care implementation fidelity studies [12, 17, 24, 29].

### Hasson’s conceptual framework for implementation fidelity

Implementation consists of adherence components (measurable) and moderating factors (non-measurable) which inform and can influence fidelity [11]. For this study, Hasson’s conceptual framework for implementation fidelity was used as it expands upon Carroll et al.’s seminal conceptual framework for implementation fidelity [11] by proposing the inclusion of context and recruitment as additional moderating factors [30]. Please see Table 1 for further definitions of adherence and moderating factors within the implementation fidelity context. Hasson’s conceptual framework has also been previously employed to assess the implementation fidelity of a pharmacist intervention [17].

**Table 1:** Implementation fidelity terms and definitions adapted from Carroll et al. [11] and Hasson [30]

Implementation fidelity terms	Definition
Adherence	The measure by which implementation fidelity is assessed. This measure determines whether the intervention was delivered as intended [11]. The higher the fidelity, the greater extent to which the intervention was delivered as intended [11]. Adherence measurements are quantifiable and comprise of the following subcategories – content, frequency, duration and coverage [11]
Moderating factors	A range of factors may impact the extent to which an intervention was implemented as intended [11]. According to Hasson, the following moderating factors have the potential to positively or negatively influence fidelity – participant responsiveness, comprehensiveness of policy description i.e. intervention complexity, facilitation strategies, quality of delivery, recruitment and context [30]. Participant responsiveness relates to how participants delivering as well as receiving the intervention perceive the intervention’s relevance and are engaged with the intervention [11, 30]. Intervention complexity relates to the description of the intervention [11] as well as the complexity of the intervention itself [30]. Facilitation strategies relates to the strategies employed to standardise and optimise implementation fidelity [11]. Quality of delivery relates to appropriate delivery of the intervention as intended [11]. Recruitment relates to the processes supporting participants to participate in the intervention [30]. Context relates to the structures, cultures and concurrent events which may impact intervention implementation [30]

### Adherence assessment

In this study, implementation fidelity adherence was assessed based upon quantitative data, consistent with previous health care implementation fidelity studies [12, 19, 25, 29]. The three quantitative data sets selected for this study related to: (1) range of OSP intervention activities delivered; (2) random sample of 10% of medication reviews assessed for quality; (3) proportion of residents who received at least one medication review. These quantitative data sets were chosen given the pragmatic nature of this study and as they provided insights into the extent of both resident and RACF level activities delivered as part of the OSP intervention.

### Moderating factors

The moderating factors influencing OSP intervention delivery were identified based upon qualitative data, consistent with previous health care implementation fidelity studies [12, 18, 24, 25, 29]. Explicit consideration of the moderating factors in this study is consistent with Bragstad et al.’s suggestion that adherence results need to be contextualised, thereby facilitating a more holistic understanding of implementation fidelity [12].

### Participants and data collection

Activities undertaken as part of OSP intervention delivery were reported by OSPs via online pharmacist diaries on the Qualtrics survey platform [31]. These activities were within the current scope of practice of pharmacists registered with the Australian Health Practitioner Regulation Agency and were categorised into the following nine activities: clinical audits, medication reviews, communication, administrative tasks, vaccination, education, quality improvement and other (including fire safety training, signing statutory declarations and other facility level activities). OSPs were also asked to maintain a written copy of medication reviews undertaken for residents.

Managers and OSPs across each of the seven intervention RACFs were invited by email to participate in semi-structured interviews with a purposive (stratified) sampling approach employed [32]. These two participant groups were selected as it was considered that they would have the most insight into OSP intervention delivery and the factors influencing delivery of this intervention. The RACF manager and OSP interview guides used in this study have been published elsewhere [33]. Interviews were audio-recorded, transcribed and deidentified to help maintain participant anonymity and confidentiality [34].

### Data analysis

The online pharmacist diaries were downloaded from Qualtrics and cleaned and checked in Microsoft Excel. The proportion of residents who received at least one medication review was determined by the number of written medication reviews provided by OSPs to the study team. A random sample of 10% of these written medication reviews were assessed for quality by two Medication Management Review Accredited Pharmacists (accredited pharmacists^2^) using a checklist adapted from Curtain’s medication review quality assessment work [35]. The medication review quality assessment approach taken in this study builds upon the Care Home Independent Prescribing Pharmacist Study (CHIPPS) wherein a random sample of pharmaceutical care plans were reviewed for appropriateness by suitable experts [36]. For this study, the Intraclass Correlation Coefficient between the two accredited pharmacists was also assessed. This approach is consistent with Gearing et al.’s recommendations regarding the use of two or more independent raters and assessment of inter-rater reliability [22]. For each of the three quantitative data sets, the study team developed an adherence scoring system, as well as an overall implementation fidelity adherence scoring system consistent with Bragstad et al.’s implementation fidelity rating system of low, medium or high [12]. Please see Additional file 1 for further details of these scoring systems. The use of these objective adherence scoring systems increases the validity and reliability of these fidelity measures, consistent with Gearing et al.’s recommendations [22].

For this study, the qualitative data was analysed using Ritchie and Spencer’s framework analysis approach [37] with data deductively mapped to applicable moderating factors described in Hasson’s conceptual framework for implementation fidelity. Co-authors contributed to data analysis and interpretation with NVivo used to support data management and assist with clear audit trail documentation [38]. Qualitative data were reported according to the Consolidated Criteria for Reporting Qualitative Research (COREQ) checklist [39]. Please see Additional file 2 for further details.

Data integration for this convergent parallel mixed methods study was undertaken at the interpretation stage with quantitative, qualitative and then integrated findings presented in this article. The mixed methods findings were reported noting Hadi et al.’s [40] recommendations to improve mixed methods research reporting for pharmacy practice researchers.

## Results

### Adherence

#### Range of OSP intervention activities delivered

Within the seven intervention RACFs, the full range of OSP intervention activities were delivered i.e. the online pharmacist diaries indicated that OSPs delivered activities in all nine activity categories. There was one exception to this with one OSP not able to offer vaccination services as they were unable to complete vaccination training due to COVID-19 restrictions affecting training availability. As such, the full range of OSP intervention activities were delivered across each of the seven intervention RACFs as illustrated in Table 2.

#### Random sample of 10% of medication reviews assessed for quality

Assessment of a random sample of 10% of written medication reviews by two accredited pharmacists using a checklist indicated that the quality of medication reviews across the seven intervention RACFs ranged from high (n = 3), medium (n = 3) through to low (n = 1) as illustrated in Table 2. The rounded mean score for the quality assessment of all written medication reviews was 3 out of 5 indicating an overall medium quality. Assessment of Intraclass Correlation Coefficient (ICC) indicated excellent reliability between the two accredited pharmacists, namely, an ICC of 0.922 (95% CI: 0.697 to 0.974).

#### Proportion of residents who received at least one medication review

The proportion of residents who received at least one medication review was compared to the PiRACF study *a priori* activity target of 70% of residents receiving at least one medication review as part of the OSP intervention. OSPs supplied 588 written medication reviews to the study team and 61.1% of residents living across the seven intervention RACFs received at least one medication review as part of the OSP intervention. In this study, using the adherence scoring system, adherence to this fidelity measure ranged from high (n = 4), medium (n = 1) through to low (n = 2), see Table 2.

#### Overall implementation fidelity rating

Based upon the three quantitative data sets, the overall implementation fidelity adherence score for the seven intervention RACFs ranged from high (n = 1), medium-high (n = 3) through to medium (n = 2) and low-medium (n = 1), as illustrated in Table 2. Overall, it appears that the OSP intervention was generally delivered with medium fidelity across the seven intervention RACFs.

**Table 2:** Adherence assessment of RACFs

RACF	Range of OSP intervention activities delivered	Random sample of 10% of medication reviews assessed for quality	Proportion of residents who received at least one medication review	Overall score
1	Yes	High	Medium	Medium – High
2	Yes	Medium	High	Medium – High
3	Yes	Medium	Low	Low – Medium
4	Yes	Medium	High	Medium – High
5	Yes	High	Low	Medium
6	Yes	High	High	High
7	Yes	Low	High	Medium

### Moderating factors

Fourteen interviews were undertaken with RACF managers (n = 7) and OSPs (n = 7 interviews with six OSPs [one OSP worked across two RACFs]). These interviews took between 38 and 163 min, with the median interview duration of 49 min for managers and 148 min for OSPs. Semi-structured interview participant details are shown in Table 3. Most pharmacists had over 10 years professional experience and five were accredited pharmacists. As such, this study was not able to determine if there may be a possible correlation between the level of pharmacist experience and intervention fidelity.

**Table 3:** Semi-structured interview participants

Profession	Number of participants	Age (years)	Gender	Professional experience (years)	Length of employment at RACF (years)	Prior aged care experience
On-site pharmacist	6*	≤ 40 (4, 67%) > 40 (2, 33%)	F (56, 83%) M (1, 17%)	≤ 5 (1, 17%) ≥ 10 (5, 83%)	≤ 1 (6, 100%)	Residential Medication Management Review experience (2, 33%) Supplying medications to RACF(s) experience (1, 17%)
RACF manager	8 managers**	≤ 50 (2, 25%) > 50 (6, 75%)	F (6, 75%) M (2, 25%)	≤ 15 (2, 25%) > 15 (6, 75%)	≤ 1 (3, 37.5%) > 1 (5, 62.5%)	≤ 4 (2, 25%) > 4 (6, 75%)

* Across the seven intervention RACFs, one OSP was interviewed at each RACF with one pharmacist employed by two RACFs

** includes characteristics of RACF manager who provided written feedback in lieu of an interview

The qualitative interviews provided insights into the following moderating factors outlined in Hasson’s conceptual framework for implementation fidelity: intervention complexity, facilitation strategies, quality of delivery, participant responsiveness and context [30]. Please see Table 4 for the moderating factors, a summary of key findings and exemplary quotes.

**Table 4:** Moderating factors with a summary of key findings & exemplary quotes

Summary of key findings	Exemplary quotes
**Intervention complexity**	
It took time to work out how to deliver the OSP intervention	*when [the OSP] first started, we never had one, so we didn’t know what to do with [the OSP] when [the OSP] started… I would say, it took us probably about three months to really get into the swing of what we needed [the OSP] to really do. (M7.1)*
Facilitator: study resource folder	*having that information [in the study resource folder] … meant that we were all coming from the same idea that we want to be accessible, reduce medications where possible and rationalise them, and improve medication management from being on site. (OSP 7)*
Barrier: OSP job description	*There were too many items on the attached Position Description provided at the commencement of [their] contract to be realistic for two days per week. (M5.1)*
	*I didn’t feel I have a very clear job description. The facility didn’t know what my job was going to be either. (OSP 1)*
**Facilitation strategies**	
Facilitator: Pharmacist support meetings	*I love the three-monthly meetings… they’ve been really helpful just to reset and refocus and get a bit of guidance what to focus on next. (OSP 6)*
**Quality of delivery**	
Generally delivered to a high standard	*Two years ago at RACF 6, we were completely non-compliant with medications; we didn’t meet the standard at all. So I’m being honest here. So in the last year, having [the OSP] here, we’ve been able to become completely compliant… having a pharmacist to go to… you can actually see the difference with medication management, [it] has improved immensely. (M6.1)*
One exception – one OSP not able to offer vaccination services	*I know [the OSP] did try to get that credential [to be able to vaccinate], but [they] couldn’t find any courses that were available. That would probably be the only thing that would’ve been quite beneficial to us. (M7.1)*
Barrier: Part-time nature of OSP role	*I’m not always here when the GPs are here and I’m not always here when the changes are made. (OSP 7)*
	*It would have made an even greater impact if she was able to work more than two days per week to allow for greater follow up. e.g. if [the OSP] sent an email on Friday, [the OSP] could not follow up the response till the following Wed, five days later. (M5.1)*
Facilitator: Prior OSP experience	*[The OSP] had already started at another facility before [they] started here… so we implemented pretty much what [they were] already doing at that other one here… And it kind of worked really well in with what we wanted to do, anyway… So within a couple of weeks, [we] were just flying. (M6.1)*
**Participant responsiveness**	
Participants mostly perceived that OSPs in RACFs added value and OSP intervention should continue	*it’s just a very valuable resource [having the OSP] that would just only complement the clinical team and the workforce within the facility. (M2.1)*
	*But if they could see their way clear to fund [an OSP], I think it’d be a good outcome for every aged care [facility]. (M1.1)*
	*So I feel everyone is used to me being here and sees value in me being here and would like me to stay. (OSP 1)*
Participants [specifically RACF staff] were responsive	*They’re all like that if [the OSP’s] here, [the OSP’s] helpful. I felt that they go to [the OSP] if they need to and ask questions if they have to. (M3.1)*
‘Missed opportunities’: Limited OSP work day availability which contributed to delays in OSP intervention being delivered as intended	*I think we've got up to up to full-steam now over the last couple of months… [but] there was missed opportunity in the beginning which was no one’s fault… [which] slowed the [initial] uptake of engagement with the GPs. (M4.1)*
‘Missed opportunities’: Perceived limited impact of OSP intervention due to the OSP (who was not accredited) not focussing on medication reviews in their part-time role and relying upon RACF management to guide delivery	*But to actually – to really justify having someone, for us to take it on a permanent basis, I probably would have a hard time trying to explain it... I can’t see any real big fundamental changes that have been made. (M7.1)*
	*I think what we missed – the opportunity there was more deep dives into specific residents like where we were having residents who are having large amounts of falls or were particularly unwell (M7.1)*
	*And I think because the facility of our size … it’s a very big job, and I think it was just a too big a job for [the OSP] to be able to do in the hours that [they were] here. (M7.1)*
	*I think it would’ve been good for the [OSP] to actually have an idea or have them have a plan of they wanted to do to support us. I think a lot of the onus was put on us. (M7.1)*
‘Missed opportunities’: OSP not being able to vaccinate	*[Their] colleague in RACF 6 was a lot more – well, in that respect, was a lot more useful because when the flu vaccinations came about, [OSP 6] administered all the flu vaccinations to the staff. (M7.1)*
‘Missed opportunities’: Perceived limited capacity of the RACF manager to work with the OSP to further optimise OSP intervention delivery	*I think the busyness distracts me a lot where I could be working more with people like [the OSP] to look at how do we improve processes and systems. But I think there’s a real opportunity there that I probably missed or [the OSP] might’ve missed where we could do more work together. (M2.1)*
	*I think it’s an invaluable service that we’re now going to lose, having an onsite pharmacy expert just, as I said, as a quick reference point then to help us with our assessment and management of residents and their medications. (M2.1)*
	*it’s sad knowing that the role’s coming to an end because I think the longer [the OSP] would be here… [the more possible it would be] to see what else [the OSP] can do that would help us in our medication management. (M2.1)*
**Context - OSP factors**	
Facilitator: OSPs who took time to establish relationships and were pro-active in informing OSP intervention delivery	*So I think that would give an incoming [OSP] a big advantage later down the track and save a lot of time, if they do establish their role and those relationships as soon as possible. (OSP 6)*
	*So basically, I had to inject myself and say, “Look, I can take that workload from you. I can do that for you. I can help with that,” and really push a little bit at the beginning to say, "Look, I am actually here to help you and make your life easier." (OSP 1)*
	*And [the OSP] also said to us, “I feel like I could be of help here.” (M1.1)*
Facilitator: Experienced accredited pharmacists	*Well, I guess being accredited really helped… I think that without that, I would have had to get into the groove of reviewing medication charts. (OSP 3)*
**Context – RACF factors**	
Facilitator: RACF managers who actively supported OSPs	*As Care Manager I worked closely with our onsite Pharmacist and gave [them] a clear list of priorities each week that we would like [them] to focus on. (M5.1)*
	*[The OSP] was sitting down in the Aged Care Funding Instrument office [initially which meant that the OSP’s] not in any flow traffic, GPs [as well as residents and staff] weren’t able to easily access [the OSP]. So, I moved [the OSP] into my office… [and] I think we did get [the OSP]… more included into the facility… [and] more probably in the middle of it. (M7.1)*
Facilitator: Positive RACF culture	*The staff here and the residents here are all lovely. The staff are really putting their residents first. The attitude is very – it’s a family, we’re looking after each other, and they’re really supportive of each other as well and I feel like that flows through to the care and encourages me to care more as well, and do my best. (OSP 1)*
Barrier: RACFs spread out over a large area with significant distance between resident dwellings	*partly because of the way it’s set up … you stay in your bubble a lot more here than at the previous [RACF] which was all one big communal space… It’s not an organic thing here. I have to actually go to the [resident dwellings in this RACF which is spread out] and meet them and talk to them and all that which is a bit different. It is a much bigger facility as well. So getting to know particular residents really, really well has been a lot harder, whereas at the last facility, there were some residents that I saw every day that I was there and got to know really, really well. (OSP 6)*
**Context – external factor**	
Barrier: COVID-19 pandemic	*Look, it [the OSP intervention] came at a really tricky time of COVID-19… [we were] so busy focusing on compliance with COVID-19 monitoring requirements. (M4.1)*
	*So there was a bit of time [due to COVID-19] where it was difficult to talk to the residents… it was stressful a bit for the staff especially, and we had to wear masks all the time for a while, and some of the residents expressed frustration and difficulty seeing their family and all that. But my role as such, I was still able to do most of my tasks, it’s just the talking to people and to the residents, that was really restrictive. And to be honest, it’s taken a while to get back out of that habit. (OSP 1)*

#### Intervention complexity

The qualitative findings suggested that it took time for OSPs and RACF managers to work out how to deliver the OSP intervention, which is not unexpected given the relative novelty of the OSP role and the complexity of the intervention. OSPs considered that the OSP intervention description outlined in the study resource folder was informative and useful in assisting them with delivering this complex intervention. However, some OSPs and RACF managers mentioned that the OSP job description was not as descriptive or instructive as it could have been to support OSP intervention delivery.

#### Facilitation strategies

A range of facilitation strategies were employed to support delivery of the OSP intervention. These included face-to-face training sessions for OSPs, inclusive of the Residential Aged Care Pharmacist: Foundation Training Program facilitated by the Pharmaceutical Society of Australia (PSA), RACF induction checklist for onboarding OSPs, study team induction meeting with individual RACF managers, as well as with individual OSPs which focussed on OSP orientation, four hour face-to-face quarterly pharmacist support meetings held at the University of Canberra, Microsoft Teams Online Forum to allow OSPs to share information and ask questions in an asynchronous manner and ad hoc individual check ins with OSPs by the study team via face-to-face visits to RACFs and emails. All OSPs attended the half day face-to-face training session and pharmacist support meetings. Overall, OSPs indicated that the facilitation strategies employed, in particular, the pharmacist support meetings, were conducive to the OSP intervention being delivered as intended.

There was however some room for improvement identified in relation to OSP training provided with two OSPs expressing an interest in more palliative care training and self-nominating to attend a Program of Experience in the Palliative Approach (PEPA) training session during the 12-month OSP intervention.

#### Quality of delivery

Most of the RACF managers interviewed indicated that the OSP intervention was generally delivered to a high standard. With regards to quality of delivery, one key exception related to one OSP who was not able to offer vaccination services to RACF staff as they were unable to complete vaccination training due to COVID-19 restrictions affecting training availability. While all other OSPs offered vaccination services to RACF staff, some OSPs were not able to undertake these services due to vaccination fridge unavailability at two RACFs and due to one RACF using an existing external contractor arranged by their parent organisation. A specific barrier to the quality of delivery identified by four OSPs and two managers related to the part-time nature of the OSP role. One OSP worked across two intervention RACFs participating in the PiRACF study and described how their OSP experience at the first RACF aided them to support OSP intervention delivery with greater ease at the second RACF. This suggested that prior OSP experience was a potential facilitator for delivering the intervention.

#### Participant responsiveness

Most OSPs and managers engaged well with delivery of the OSP intervention with most participants perceiving that OSPs in RACFs added value and that the OSP intervention should be continued in the future. The qualitative findings also suggested that others, such as RACF staff, were responsive to delivery of the OSP intervention.

However, three RACF managers did identify barriers which contributed to OSP intervention ‘missed opportunities’ within their RACFs. One RACF manager described that the OSP’s limited work day availability and being unwell at the start of the intervention contributed to delays with the OSP establishing relationships with General Practitioners. This meant that it took additional time for the OSP intervention to be optimally delivered.

A second RACF manager perceived limitations on the impact of the OSP intervention due to the OSP at their RACF, who was not accredited, not focussing on medication reviews in the part-time role with limited hours available. The manager also highlighted that the OSP’s reliance upon RACF management to guide OSP intervention delivery meant that the OSP intervention may not have been optimally delivered.

The one exception where an OSP was not able to vaccinate also presented a missed opportunity within that RACF. The manager at another RACF outlined their limited capacity to engage with the OSP to identify opportunities to work together to further optimise delivery of the OSP intervention.

#### Context

Based upon the semi-structured interviews with RACF managers and OSPs, OSP factors, RACF factors and an external factor may have influenced the fidelity of OSP intervention delivery.

#### OSP factors

The qualitative findings suggested that RACFs with OSPs who took the time to establish relationships and were pro-active in informing OSP intervention delivery potentially increased the likelihood of the OSP intervention being delivered as intended. Three OSPs also considered that their accredited pharmacist status and experience conducting medication reviews may have helped them to deliver the OSP intervention.

#### RACF factors

Semi-structured interview participants described how RACF leadership and culture affected OSP intervention delivery. RACFs with management who consistently took the time to work with OSPs to inform OSP intervention delivery and actively supported OSPs within their respective RACFs potentially increased the likelihood of the OSP intervention being delivered as intended. As might be expected, there was a sense that RACFs with a positive culture focussed on collaboration and patient-centred care also increased the likelihood of delivering the OSP intervention as intended.

RACFs spread out over a large area with significant distance between resident dwellings was perceived as a potential barrier to OSP intervention delivery. One OSP who worked across two RACFs perceived that an RACF with this physical environment, as compared to one with a main RACF building, potentially decreased the likelihood of delivering the OSP intervention as intended. It appeared to be more difficult for an OSP to establish and maintain connections with health care team members and residents in the more spread out RACF physical environment without the OSP making a concerted effort to overcome this barrier.

#### External factor

One external factor was identified as a potential barrier to OSP intervention delivery, namely the COVID-19 pandemic. As the OSP intervention commencement was staggered from April 2020 – January 2021, this meant that the impact of COVID-19 varied across intervention RACFs, though there were commonalities in relation to an overall increased workload on RACF staff and managers trying to mitigate the risk of COVID-19 for residents and RACF staff. One OSP who commenced in 2020 described how the COVID-19 pandemic initially limited their capacity to engage with residents, family members and RACF staff and that it then took some time to re-engage with them.

### Integrated findings

The quantitative findings indicated that the OSP intervention was generally delivered as intended with a range of fidelity from low-medium to high and an overall finding of medium fidelity across the seven intervention RACFs. These adherence scores were based upon three quantitative data sets: (1) range of OSP intervention activities delivered; (2) random sample of 10% of medication reviews assessment for quality; (3) proportion of residents who received at least one medication review. Across the seven intervention RACFs, the full range of activities were delivered, there was an overall medium quality assessment of medication reviews and 61.1% of residents received at least one medication review (as compared to the PiRACF study *a priori* activity target of 70%). The qualitative findings illustrated that the facilitation strategies in place supported OSP intervention delivery, that participants were mostly responsive to the OSP intervention and that the quality of delivery was generally perceived to be of a high standard. Importantly, missed opportunities were identified by three RACF managers which potentially impacted OSP intervention delivery. These included: OSP work day availability which contributed to delays in the OSP intervention being optimally delivered; perceived limited OSP intervention impact due to a non-accredited OSP who was not able to focus on medication reviews during their part-time role whom was also not pro-active in guiding OSP intervention delivery; one OSP not being able to vaccinate; and perceived limited capacity of a RACF manager to work with the OSP to further optimise OSP intervention delivery. Potential barriers (including the part-time OSP role, COVID-19 pandemic, RACFs spread out over a large area with significant distance between resident dwellings) and facilitators (including the study resource folder, pharmacist support meetings, OSPs who took time to establish relationships, experienced accredited pharmacists, RACF managers who actively supported OSPs and worked with them, positive RACF culture) were also identified. Overall, it appears that the medium fidelity of OSP intervention delivery was influenced, either positively or negatively, by a range of moderating factors.

## Discussion

This mixed methods study used an established conceptual framework to understand the extent of implementation fidelity and the moderating factors influencing the implementation fidelity of OSP intervention delivery. Prior to the roll out of OSPs within Australian RACFs, it is important to understand whether the OSP intervention can be delivered as intended and what factors moderated intervention delivery. This study found that OSP intervention delivery was implemented with overall medium fidelity across the seven intervention RACFs. Furthermore, several moderating factors contributed to this fidelity, consistent with other health care implementation fidelity studies [25, 29]. The facilitation strategies in place were conducive to delivery of the OSP intervention as intended. Participants were generally responsive and most participants considered that the quality of the intervention was to a high standard. Contextual factors (OSP, RACF and external i.e. COVID-19 pandemic) and the complexity of the intervention itself also impacted OSP intervention delivery. A new and novel contribution of this study was that it identified potential barriers and facilitators to successful OSP intervention delivery in Australian RACFs. More pharmacists with prior OSP experience would likely further support implementation of this intervention in the future.

This study’s finding of an overall medium fidelity rating is relatively comparable to other studies assessing the implementation fidelity of pharmacist interventions in other health care settings [17–19]. Sluggett et al.’s mixed method process evaluation of the SImplification of Medications Prescribed to Long-tErm care Residents (SIMPLER) study undertaken in Australian RACFs concluded that their intervention was also generally delivered as intended [16]. The lack of clearly defined adherence measures in health care settings has been previously identified [29] and further consideration of suitable adherence measures to help with standardisation of implementation fidelity assessment for future pharmacist RACF intervention studies is encouraged.

The findings of this study indicated that there were moderating factors which informed OSP intervention delivery. Consistent with van der Laan et al.’s study [18], which assessed implementation fidelity of an intervention in Dutch community pharmacies, this study’s participants suggested that the intervention generally added value with participants mostly responsive to intervention delivery. Facilitation strategies, inclusive of training provided to pharmacists in this study were important for successful intervention delivery, similar to other pharmacist intervention studies which have assessed implementation fidelity [17, 18]. Akin to En-Nasery-de Heer et al.’s mixed method study [17] which explored implementation fidelity of a pharmacist-led intervention involving Dutch community and hospital pharmacists, this study also identified potential barriers for intervention delivery in relation to intervention complexity and time constraints.

According to Hasson, the more clearly defined and described the intervention, the higher the likelihood of fidelity [30]. As such, it is recommended that future efforts to adopt OSPs in Australian RACFs include additional documentation supportive of OSP intervention delivery, particularly in relation to operationalising the OSP intervention which could potentially include initial and ongoing promotion of the OSP role. In addition, tailored support to OSPs and RACF management to facilitate more consistent delivery of the OSP intervention such as through development of additional checklists and guidance documents may be beneficial. Further exploration of the extent of OSP full-time equivalent employment required, particularly within RACFs spread out over a large area with significant distance between resident dwellings, to support effective delivery of the OSP intervention is required.

OSPs highly valued the pharmacist support meetings which enabled them to share their experiences and insights with each other. While the role of pharmacists in Australia has expanded beyond community and hospital pharmacy into Aboriginal Community Controlled Health Organisations (ACCHOs) [41], General Practices [42] and now into RACFs [10], there are limited opportunities available for these pharmacists to meet and connect with other pharmacists working in similar roles. Current avenues which OSPs could access to connect with other non-community and non-hospital pharmacists are limited to the Pharmaceutical Society of Australia’s Interdisciplinary Team-Based Care Community of Speciality Interest [43]. It is recommended that there be further consideration of options to support future OSPs working in Australian RACFs to sustainably connect with each other now and into the future.

In anticipation of the roll out of OSPs within Australian RACFs it would be highly beneficial to consider the overall educational needs of pharmacists commencing in this recently created role. As there is sparse literature on the educational needs of OSPs working in RACFs, in time, when there is a body of experts with expertise on OSPs within RACFs, it is strongly encouraged that consensus be reached on OSP educational needs through undertaking either a Nominal Group Technique or Delphi Technique [44]. Benson et al.’s Delphi study provides an instructive templar on Australian General Practice Pharmacist educational needs [45]. In the interim, it may be useful for OSP educational needs to be guided by the recommended qualifications, skills and training requirements outlined for pharmacists integrated into ACCHOs, General Practices and RACFs within the PSA Pharmacists in 2023: Roles and Remuneration document [46], alongside completion of the PSA Residential Aged Care Pharmacist: Foundation Training Program (or equivalent). Further exploration of the minimum level and extent of pharmacist experience required to effectively support delivery of the OSP intervention is needed.

As identified by Tait et al., pharmacists can contribute to interprofessional collaborative care for older people living in the community and RACFs as they near their end of life [47]. As such, including additional palliative care training to develop OSP skills in end-of-life medication management discussions with residents and family members is strongly encouraged. At a minimum, it is recommended that OSPs should complete the Palliative Care Online Training developed based upon the palliAGED online resources [48] and attend PEPA training session to further understand the use of medications at end-of-life and become more confident in discussing end-of-life care with residents, family members and RACF staff. The potential role of OSPs in supporting residents with end-of-life care needs alongside health care team members and family members, should also be further explored.

Consistent with Choi et al.’s mixed method study [23] which explored implementation fidelity of a person-centred complex intervention in South Korean nursing homes, RACF culture appeared to impact intervention delivery in this study. Given that the OSP intervention was implemented within an existing RACF health care team and culture, we would reaffirm Palmer et al.’s recommendation that organisation readiness be assessed before implementing interventions [24]. One tool which could be employed before implementation of an OSP intervention within Australian RACFs is the Organisational Readiness to Change Assessment (ORCA) [49]. The ORCA tool could potentially help to increase implementation fidelity with its specific consideration of contextual measures (such as staff culture, senior leadership culture) and facilitator measures (such as project communication, planning and team roles to support intervention delivery) [49].

This is the first study that has evaluated the implementation fidelity of an OSP intervention delivered within Australian RACFs. This study demonstrated that the OSP intervention could be delivered with medium fidelity across seven intervention RACFs and reaffirmed the importance of understanding moderating factors which could help to identify barriers or facilitators to successful OSP intervention delivery.

Policy makers, primary health networks, peak pharmacy organisations, researchers, health professionals and RACF management are strongly encouraged to consider the findings of this study and recommendations made prior to the roll out of OSPs in Australian RACFs. These study findings could help inform future efforts to address potential barriers and enhance potential facilitators for successful adoption of OSPs in real world RACFs.

### Strengths and limitations

Strengths of this study was the use of mixed methods study design, an existing implementation fidelity framework and development of objective fidelity scoring systems.

Study limitations related to the qualitative findings not being generalisable to other non-metropolitan RACFs. As direct observations by the study team were not conducted and the quantitative activities data was self-reported, potential data accuracy issues may exist [50].

## Conclusion

This mixed methods study concluded that the OSP intervention was generally delivered as intended across the seven intervention RACFs with an overall medium fidelity rating. OSP intervention delivery was affected by a range of moderating factors, specifically, intervention complexity, facilitation strategies, quality of delivery, participant responsiveness and context. A number of potential barriers and facilitators to successful OSP intervention delivery were also identified. The findings of this study have important implications for the roll out of OSPs in Australian RACFs and further OSP research studies.

### Electronic supplementary material

Below is the link to the electronic supplementary material.

Supplementary Material 1

Supplementary Material 2

## Data Availability

The deidentified quantitative data used to support the findings of this study are available from the corresponding author upon request. The qualitative data is not available as it is possible that this data could be re-identified.

## Footnotes

1 Informed upon the Australian Government Department of Health and Aged Care’s Glossary For The Guiding Principles and User Guide, published in 2023. For the purposes of this study, medication management is defined as the multiple steps associated with the provision of medication from prescribing through to administration and monitoring of use, with the resident at the centre of this often complex process.

2 A pharmacist registered with the Australian Health Practitioner Regulation Agency needs to undertake additional training to become an accredited pharmacist. The Australian Government provides funding for accredited pharmacists to conduct medication management reviews for people living in the community and in RACFs, following referral from the patient’s (or resident’s) General Practitioner.
