# Supplementary material for: Assessing implementation fidelity of an on-site pharmacist intervention within Australian residential aged care facilities: A mixed methods study
Source: BMC Health Serv Res. 2023 Oct 27;23:1166. doi: 10.1186/s12913-023-10172-9 (PMC10604517; doi:10.1186/s12913-023-10172-9)
Supplement: Supplementary file 1 — Supplementary Material 1 [file 12913_2023_10172_MOESM1_ESM.docx]

**Additional file 1: Adherence scoring systems**

*Range of OSP intervention activities delivered – scoring system*

| **Rating** | **Activities undertaken** |
| --- | --- |
| Yes | Full range of OSP activities* delivered |
| No | Full range of OSP activities* not delivered |

* Coverage across all OSP intervention activities inclusive of clinical audits, medication reviews, communication, administrative tasks, vaccination, education, quality improvement and other

*Random sample of 10% of medication reviews assessed for quality – scoring system*

| **Rating** | **Quality assessment**** |
| --- | --- |
| High | > 4 |
| Medium | 3 |
| Low | < 2 |

** rounded mean score

*Proportion of residents who received at least one medication review – scoring system*

| **Rating** | **Proportion compared to *a priori* activity target of 70%** |
| --- | --- |
| High | >70% |
| Medium | 69 – 46% |
| Low | < 45% |

*Overall implementation fidelity adherence – scoring system*

| **Quantitative data set 1** | **Quantitative data set 2** | **Overall score***** |
| --- | --- | --- |
| High | High | High |
| High | Medium | Medium – High |
| Medium | Medium | Medium |
| High | Low | Medium |
| Medium | Low | Low – Medium |
| Low | Low | Low |

***If the full range of OSP activities are all delivered (Yes), Overall score to remain the same rating. If the full range of OSP activities are not all delivered (No), Overall score to be rated down from High to Medium-High, Medium-High to Medium, Medium to Low-Medium, Low-Medium to Low
