# Supplementary material for: Assessing implementation fidelity of an on-site pharmacist intervention within Australian residential aged care facilities: A mixed methods study
Source: BMC Health Serv Res. 2023 Oct 27;23:1166. doi: 10.1186/s12913-023-10172-9 (PMC10604517; doi:10.1186/s12913-023-10172-9)
Supplement: Supplementary file 2 — Supplementary Material 2 [file 12913_2023_10172_MOESM2_ESM.docx]

**Additional file 2: Qualitative findings reported according to the Consolidated Criteria for Reporting Qualitative Research (COREQ) checklist (1)**

No Item Guide questions/description

**Domain 1: Research team and reflexivity**

*Personal Characteristics*

1. Interviewer/facilitator Which author/s conducted the interview or focus group? Lead author with prior qualitative experience

2. Credentials What were the researcher’s credentials? *E.g. PhD, MD* Bachelor of Pharmacy, Master of Public Health and PhD candidate

3. Occupation What was their occupation at the time of the study? PhD candidate

4. Gender Was the researcher male or female?

Female

5. Experience and training What experience or training did the researcher have? Master of Public Health with prior qualitative experience

*Relationship with participants*

6. Relationship established Was a relationship established prior to study commencement?

This did not occur with RACF managers, but the lead author had met on-site pharmacists (OSPs) at a face-to-face pharmacist support meeting prior to conducting interviews

7. Participant knowledge of the interviewer

What did the participants know about the researcher? *e.g. personal goals, reasons for doing the research*

Participants were aware that the researcher was a PhD candidate

8. Interviewer characteristics What characteristics were reported about the interviewer/facilitator? e*.g. Bias, assumptions, reasons and interests in the research topic*

Participants were aware that the interviewer was part of the PiRACF study team and a PhD candidate prior to interviews commencing

**Domain 2: study design**

*Theoretical framework*

9. Methodological orientation & Theory What methodological orientation was stated to underpin the study*? e.g. grounded theory, discourse analysis, ethnography, phenomenology, content analysis*

This study was informed by a pragmatism research design, underpinned by a qualitative descriptive approach

*Participant selection*

10. Sampling How were participants selected? e.g. purposive, convenience, consecutive, snowball

A purposive (stratified) sampling approach was used

11. Method of approach How were participants approached? e*.g. face-to-face, telephone, mail, email*

Participants were recruited by the lead author via email

12. Sample size How many participants were in the study?

14

13. Non-participation How many people refused to participate or dropped out? Reasons?

Not applicable

*Setting*

14. Setting of data collection Where was the data collected? *e.g. home, clinic, workplace* Interviews were held online

15. Presence of non-participants Was anyone else present besides the participants and researchers?

No non-participants were present during interviews

16. Description of sample What are the important characteristics of the sample? *e.g. demographic data, date*

Participants consisted of at least one RACF manager and at least one OSP from each of the 7 intervention sites. Interviews were conducted between April – October 2021

*Data collection*

17. Interview guide Were questions, prompts, guides provided by the authors? Was it pilot tested?

An interview guide was used which was initially pilot tested with a nurse and pharmacist with aged care experience

18. Repeat interviews Were repeat interviews carried out? If yes, how many? No repeat interviews were conducted, although the OSP interviews were conducted in two sessions to minimise participant burden

19. Audio/visual recording Did the research use audio or visual recording to collect the data?

Audio recordings

20. Field notes Were field notes made during and/or after the interview or focus group?

To support ongoing reflexivity, the lead author completed a templated contact summary sheet after each interview to contemporaneously document the researcher’s reflections and maintained a reflexive diary to record the research process and reflections

21. Duration What was the duration of the interviews or focus group?

Interviews ranged from 38 to 163 minutes in duration

22. Data saturation Was data saturation discussed?

The interview sample size was predetermined taking a pragmatic approach to available time and resourcing (2). While a recent study has suggested that data saturation can be achieved with between 9-17 interviews (3), the use of data saturation to inform interview sample sizes for non-grounded theory qualitative research remains debatable (2, 4)

23. Transcripts returned Were transcripts returned to participants for comment and/or correction?

No

**Domain 3: analysis and findings**

*Data analysis*

24. Number of data coders How many data coders coded the data?

The leader author coded the data

25. Description of the coding tree Did authors provide a description of the coding tree?

No

26. Derivation of themes Were themes identified in advance or derived from the data?

Themes were derived from the data using framework analysis, inclusive of the development of a coding framework and identification of themes. Data was then deductively mapped to applicable moderating factors described in Hasson’s conceptual framework for implementation fidelity (5)

27. Software What software, if applicable, was used to manage the data? NVivo was used

28. Participant checking Did participants provide feedback on the findings? No

*Reporting*

29. Quotations presented Were participant quotations presented to illustrate the themes/findings? Was each quotation identified? *e.g. participant number*

Yes, quotes identified by participant number were presented to illustrate the findings

30. Data and findings consistent Was there consistency between the data presented and the findings?

Yes

31. Clarity of major themes Were major themes clearly presented in the findings?

Yes

32. Clarity of minor themes Is there a description of diverse cases or discussion of minor themes?

Yes

References

1. Tong A, Sainsbury P, Craig J. Consolidated criteria for reporting qualitative research (COREQ): a 32-item checklist for interviews and focus groups. International Journal for Quality in Health Care. 2007;19(6):349-57.

2. Doyle L, McCabe C, Keogh B, Brady A, McCann M. An overview of the qualitative descriptive design within nursing research. Journal of Research in Nursing. 2019;25(5):443-55.

3. Hennink M, Kaiser BN. Sample sizes for saturation in qualitative research: A systematic review of empirical tests. Social Science & Medicine. 2021:114523.

4. Malterud K, Siersma VD, Guassora AD. Sample Size in Qualitative Interview Studies: Guided by Information Power. Qualitative Health Research. 2016;26(13):1753-60.

5. Hasson H. Systematic evaluation of implementation fidelity of complex interventions in health and social care. Implementation Science. 2010;5:67.
